# Lysosomal-Associated Protein Transmembrane 4 Beta-35 Overexpression Is a Novel Independent Prognostic Marker for Gastric Carcinoma

**DOI:** 10.1371/journal.pone.0118026

**Published:** 2015-02-17

**Authors:** Luying Liu, Xuefang Xu, Lili Jing, Gengyin Zhou, Zhang Cao, Yanchun Han, Rouli Zhou

**Affiliations:** 1 Department of Pathology, Binzhou Medical University, Yantai, Shandong Province, China; 2 Department of Pathology and Pathophysiology, School of Medicine, Shandong University, Jinan, Shandong Province, China; 3 Department of Pathology, Yantai Affiliated Hospital of Binzhou Medical University, Yantai, Shandong Province, China; 4 Department of Cell Biology and Genetics, School of Basic Medical Sciences, Peking University, Beijing, China; Peking University Cancer Hospital and Institute, CHINA

## Abstract

**Objective:**

The purpose of this work was to analyze the relationships between the expression status of Lysosomal-associated protein transmembrane-4 beta 35 (LAPTM4B-35) in cancerous tissues and clinicopathological characteristics and prognosis of the patients with gastric carcinoma (GC).

**Methods:**

The GC samples from 157 patients in a discovery cohort and 148 patients in a testing cohort with follow-up data were used to validate the feasibility of expression of LAPTM4B-35 protein in predicting GC prognosis. Immunohistochemical staining was used to determine the expression of LAPTM4B-35 protein in precancerous gastric lesions and gastric carcinomas. The correlation between the expression of LAPTM4B-35 and clinicopathologic characteristics of patients with gastric carcinoma was analyzed using chi-square test. Univariate and multivariate analyses were performed to determine the association between LAPTM4B-35 expression and prognosis.

**Results:**

LAPTM4B-35 expression was increased steadily in sequential stages of precancerous gastric lesions. Positive LAPTM4B-35 expression was more frequently detected in patients with distant metastasis (*P* = 0.023) and III+IV TNM stages (*P* = 0.042) in the discovery cohort. Kaplan-Meier survival curves and univariate analysis showed that expression of LAPTM4B-35 had a significant impact on overall survival of patients with gastric carcinoma in discovery cohort (*P*<0.001) and testing cohort (*P =* 0.001). LAPTM4B-35 expression was an independent prognostic indicator for the overall survival of patients with gastric carcinoma in both cohorts.

**Conclusions:**

The present research demonstrated that LAPTM4B-35 over-expression was an independent factor in gastric carcinoma prognosis. LAPTM4B gene may be a useful target of interventions slowing the progression of precancerous gastric lesions and a new therapy method to improve the prognosis of gastric carcinoma.

## Introduction

Gastric carcinoma (GC) was a very common cancer worldwide with high mortality rate. Over 70% of new GC cases and deaths occurred in developing countries, especially in East Asia. Diagnosed at later stages and accepted inappropriate therapy were main causes of the high mortality rate of GC [1]. Molecular and genetic alterations underlying the initiation, progression and metastasis of GC made it possible to find effective markers to predict the progression and prognosis of precancerous gastric lesions and GC [2, 3]. According to these researches, interventions to slow the progression of precancerous gastric lesions and appropriate therapeutic facilities and drugs applied according to these researches might reduce the incidence of GC and improve the prognosis of GC. But the exact molecular mechanisms underlying gastric carcinogenesis and GC progression were not fully understood until now.

Lysosomal-associated protein transmembrane-4 beta (LAPTM4B) gene located at chromosome 8q22 with seven exons separated by six introns [4]. LAPTM4B gene encoded two proteins with different molecular weight, 35 kDa (named LAPTM4B-35) and 24 kDa (named LAPTM4B-24) [5, 6]. LAPTM4B-35 protein, but not LAPTM4B-24, was up-regulated in a wide range of cancers including breast carcinoma [7], pancreatic carcinoma [8], ovarian carcinoma [9, 10, 11], colon carcinoma [12], hepatocellular carcinoma [13, 14, 15], extrahepatic cholangiocarcinoma [16], cervical carcinoma [17], endometrial carcinoma [18] and gallbladder carcinoma [19]. LAPTM4B was considered to be a putative novel oncogene. Previous reports indicated that LAPTM4B-35 over-expression increased cell growth and proliferation, and promoted the progression of cancer cells towards highly invasive and metastatic stages [20, 21, 22, 23]. The mechanisms was also elucidated including activation of proto-oncogenes such as c-myc, c-fos and c-jun, up-regulation of cell cycle regulators such as cyclin D1 and cyclin E [21, 22], resistance to apoptosis, activation of PI3K/AKT signaling pathway [23], promotion autophagy [24, 25] and modulating molecules associated with degradation of extracellular matrix [26]. In the carcinomas mentioned above, over-expression of LAPTM4B-35 was closely correlated with worse prognosis. However, there were no systematic studies in expression status and significance of LAPTM4B-35 in GC and precancerous gastric lesions.

In the present research, we detected LAPTM4B-35 expression status in precancerous gastric lesions and gastric carcinomas by immunohistochemical staining. The purpose of our study was to investigate the relationships between expression of LAPTM4B-35 and the clinicopathological characteristics and prognosis of the patients with GC. We hypothesize LAPTM4B may be a useful marker to predict the progression of precancerous gastric lesions and the prognosis of patients with GC.

## Materials and Methods

### Patients

We collected a discovery cohort including 157 patients from the Affiliated Hospital of Binzhou Medical University between 2004 and 2007, and a testing cohort including 148 patients from the Yantai Affiliated Hospital of Binzhou Medical University between 2003 and 2007. All patients were diagnosed with gastric adenocarcinoma and received radical gastrectomy in the Surgical Department. There were 119 males and 38 females with a mean age of 57.8 years (range, 31–78 years) in discovery cohort, and there were 98 males and 50 females with a mean age of 57.6 years (range, 25–82 years) in testing cohort. The clinicopathological features of patients in two cohorts, including age, sex, tumor size, histopathological differentiation, TNM staging, Lauren type, vessel permeation, lymph node metastasis and distant metastasis were summarized in Table 1 and Table 2. None of the patients received systemic chemotherapy, immunotherapy or radiotherapy before and after surgery. Normal gastric mucosa (10 cases), mucosa diagnosed as chronic atrophic gastritis without intestinal metaplasia and dysplasia (10 cases), chronic gastritis with intestinal metaplasia (10 cases) and chronic gastritis with dysplasia (10 cases) were obtained from gastroscope biopsy samples. All patients consented in writing to participate in this study. This project was approved by the Ethics Committee of Binzhou Medical University before performance.

**Table 1:** Association between LAPTM4B-35 expression and clinicopathological features of patients with gastric carcinoma in discovery cohort.

Clinicopathological features	Case no.	LAPTM4B-35 Expression		
		Negative	Positive	P
Age(y)				0.393*
≦55	48	6	42	
>55	109	9	100	
Sex				0.999*
Male	119	12	107	
Female	38	3	35	
Tumor size				0.099#
<5cm	63	9	54	
≧5cm	94	6	88	
Differentiation				0.707#
Well + Moderate	70	6	64	
Poor	87	9	78	
TNM stage				0.042*
I+II	30	6	24	
III+IV	127	9	118	
Lauren type				0.690#
Intestinal type	54	6	48	
Diffuse type	79	6	73	
Mixed type	24	3	21	
Vessel permeation				0.761*
Absent	43	3	40	
Present	114	12	102	
Lymph nodes metastasis				0.444*
Absent	22	3	19	
Present	135	12	123	
Distant metastasis				0.023*
M0	119	15	104	
M1	38	0	38	
Survival time				<0.001*
≧60 months	44	12	32	
<60 months	113	3	110	

LAPTM4B: Lysosomal-associated protein transmembrane-4 beta

#: Pearson chi-square test

*: Fisher’s exact test.

**Table 2:** Association between LAPTM4B-35 expression and clinicopathological features of patients with gastric carcinoma in testing cohort.

Clinicopathological features	Case no.	LAPTM4B-35 Expression		
		Negative	Positive	P
Age(y)				0.775#
≦55	51	5	46	
>55	97	11	86	
Sex				0.739#
Male	98	10	88	
Female	50	6	44	
Tumor size				0.819#
<5cm	70	8	62	
≧5cm	78	8	70	
Differentiation				0.930#
Well + Moderate	57	6	51	
Poor	91	10	81	
TNM stage				0.187#
I+II	52	8	44	
III+IV	96	8	88	
Lauren type				0.840#
Intestinal type	75	8	67	
Diffuse type	48	6	42	
Mixed type	25	2	23	
Vessel permeation				0.113#
Absent	65	10	55	
Present	83	6	77	
Lymph nodes metastasis				0.362*
Absent	38	6	32	
Present	110	10	100	
Distant metastasis				0.226*
M0	129	16	113	
M1	19	0	19	
Survival time				<0.001#
≧60 months	52	12	40	
<60 months	96	4	92	

LAPTM4B: Lysosomal-associated protein transmembrane-4 beta

#: Pearson chi-square test

*: Fisher’s exact test

### Specimen characteristics

Tumor specimens were obtained at the time of surgery. A small sample of carcinoma tissue was collected and frozen in liquid nitrogen. All the tissue blocks got from specimens were fixed in 10% neutral buffered formalin solution, embedded in paraffin and stored at room temperature. 4μm thick sections were prepared for immunohistochemical staining. Four frozen fresh GC tissue samples were used in the Western blot analysis.

### Assay Methods

**Immunohistochemical staining.** Immunohistochemistry was used to detect the presence of LAPTM4B-35 proteins in specimens. The prepared 4μm sections were deparaffinized in xylene, rehydrated in a graded ethanol (100%, 95%, 80% and 75%) and washed in phosphate buffered saline (PBS). Endogenous peroxidase activity was blocked by incubation for 10 minutes in 3% H_2_O_2_. After antigen retrieval performed in an autoclave for 3 minutes (10 mM citrate buffer, pH 6.0), the sections were incubated overnight at 4°C with the primary antibody at a dilution of 1:50. The rabbit anti-human polyclonal antibody (named LAPTM4B-N_1–99_-pAb, against the N-terminus of LAPTM4B-35) [27], was kindly provided by Prof. Rouli Zhou. Washed with PBS for three times, the polyperoxidase-anti-rabbit IgG (Polymer Detection System for Immuno-Histological Staining, GBI, Mukilteo, WA, USA) was linked to the primary antibody. Colour development was performed by presenting peroxidase activity with 3,3’-daiminobenzidine tetrahydrochloride (DAB). The slides were counterstained with haematoxylin and viewed under light microscope. Positive and negative controls were routinely used. Normal nonimmunone rabbit serum was used to replace primary antibody to confirm that the brown signal was presented for the immunoreactivity between special antibody and antigen.

**Immunohistochemical staining assessment and scoring.** The brown signal of LAPTM4B-35 staining was mainly located in the cytoplasm or on the cell membrane. The extent of LAPTM4B-35 staining was divided to three grades. 0: no brown signal or the brown signal was found in less than 10% epithelial cells or cancer cells on the slides; 1: 11∼50% epithelial cells or cancer cells were observed with brown signal; 2: brown signal was found in more than 50% epithelial cells or cancer cells. The intensity of LAPTM4B-35 staining was divided into three grades too: no staining = 0; weak = 1; strong = 2. The expression of LAPTM4B-35 for each slide was finally separated into two groups (negative group and positive group) according to the extent grade plus the intensity grade. Samples with staining score of 0 or 1 were defined as “negative” and samples with staining score of 2, 3 or 4 were defined as “positive”. All the slides were examined by three experienced pathologists, blinded to clinical data.

**Western blotting analysis.** Frozen tumor specimens were thawed and homogenized in lysis buffer containing 1% Triton X-100 with protease inhibitor. The mixtures were centrifuged at 12,000g for 20 min at 4 C, and the supernatant was obtained. The resulting proteins (50μg per lane) were separated in a 12% sodium dodecyl sulfate (SDS)-polyacrylamide gel (PAGE) and transferred to a PVDF membrane (Millipore, Bedford, MA, USA). Membranes were blocked with 5% nonfat dry milk in Tris-buffered saline (TBS) and then incubated overnight with rabbit anti-human LAPTM4B-35 polyclonal antibodies (LAPTM4B-N_1–99_-pAb, 1:300 dilution) at 4°C. After washing with Tris-buffered saline with Tween (TBST), the PVDF membrane was incubated with diluted horseradish peroxidase-conjugated goat anti-rabbit IgG for 1h at room temperature. The blots were detected using the Amersham ECL (Enhanced Chemiluminescence) Western Blotting System. Membranes were then washed with stripping solution for 1h and treated as described above but with rabbit anti-human β tubulin polyclonal antibodies (1:3,000 dilution; Santa Cruz biotechnology, Santa Cruz, CA, USA) as an internal control.

### Study design

We retrospectively analysed tumor samples from patients with primary invasive gastric carcinoma. All patients were followed up until December 2013 with a minimum 5 years. Survival time was defined to be the period of time in months from the date of diagnosis to the date of death from GC.

### Statistical Analysis Methods

Statistical analyses were performed with SPSS 19.0 software. Pearson chi-square test was used to compare the distributions of clinicopathologic characteristics of cases with positive expression of LAPTM4B-35 and cases with negative expression of LAPTM4B-35. Linear-by-Linear Association chi-square test was used to assess trends in LAPTM4B-35 expression by gastric disorders. Overall survival was analyzed by the Kaplan-Meier method. Log-rank test in univariate analysis was used to estimate different impacts on survival between cases with positive LAPTM4B-35 expression and those with negative LAPTM4B-35 expression. The independent influence of each variable on overall survival was analyzed by the multivariate analysis of Cox proportional hazard model. The analyses were adjusted simultaneously for the age, sex, tumour size, differentiation, TNM stage, vessel permeation and lymph nodes metastasis. We used a stepwise variable selection procedure to identify independent factors prognostic for survival, and variables were removed using backward elimination according to a selection stay criterion of 0.05.

## Results

### Expression of LAPTM4B-35 in normal gastric mucosa, chronic atrophic gastritis, intestinal metaplasia, dysplasia and gastric carcinoma by immunohistochemical staining

**Fig 1:**
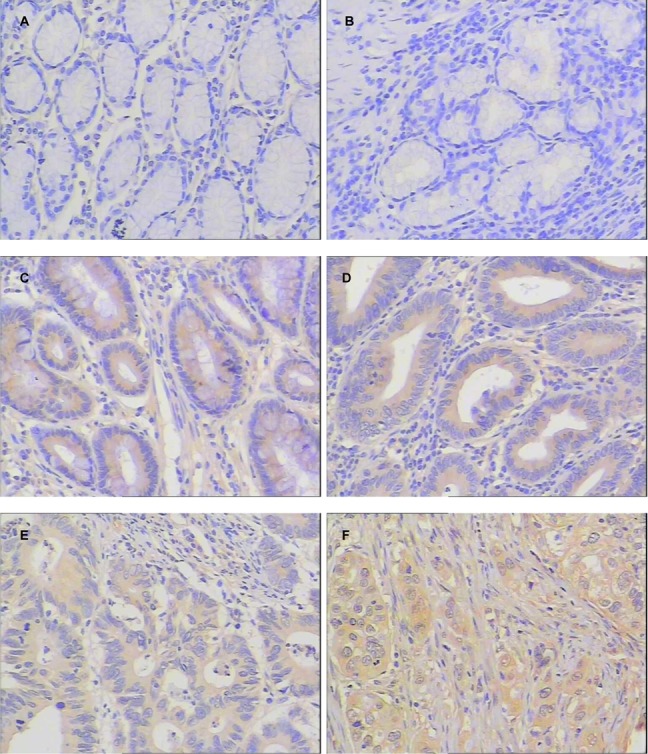
LAPTM4B-35 expression in normal gastric mucosa, precancerous gastric lesions and gastric carcinoma by immunohistochemical staining. (A) Normal gastric mucosa (score: 0+0, negative). (B) Chronic atrophic gastritis without intestinal metaplasia and dysplasia (score: 0+0, negative). (C) Intestinal metaplasia (score: 1+2, positive). (D) Dysplasia (score: 1+2, positive). (E) Moderate differentiated gastric carcinoma (score: 1+2, positive). (F) Poorly differentiated gastric carcinoma (score: 2+2, positive). Original magnification, ×200.

The LAPTM4B-35 immunohistochemical staining was mainly localized in the cytoplasm or on the cell membrane of immunoreactive cells. According to the immunohistochemical staining assessment and scoring criterion, all the normal gastric mucosa and mucosa diagnosed as chronic atrophic gastritis (without intestinal metaplasia and dysplasia) showed negative expression of LAPTM4B-35. There were 3 cases with LAPTM4B-35 positive expression among the 10 cases diagnosed as chronic gastritis with intestinal metaplasia, and 6 cases with LAPTM4B-35 positive expression among the 10 cases diagnosed as chronic gastritis with dysplasia. Among the cases with GC, intestinal metaplasia was observed in the adjacent mucosa to cancer tissues in 20 cases, and 11 cases showed positive expression of LAPTM4B-35 in intestinal metaplasia glands. In the discovery cohort (157 cases with GC), 142 (142/157, 90.4%) cases showed positive expression of LAPTM4B-35 in cancer tissues. Representative examples of LAPTM4B-35 staining were shown in Fig. 1.

The expression of LAPTM4B-35 increased steadily from 0 in normal gastric mucosa and chronic atrophic gastritis (without intestinal metaplasia or dysplasia) to 30% in chronic gastritis with intestinal metaplasia, 55% in intestinal metaplasia beside cancer tissues, 60% in dysplasia, peaking at 90.4% in GC (Table 3).

**Table 3:** Expression of LAPTM4B-35 in normal gastric mucosa, CAG, IM, IM beside GC, dysplasia and gastric carcinoma.

Gastric lesions	Cases	LAPTM4B-35 Expression		
		Negative	Positive	*P* Value #
Normal gastric mucosa & CAG	20	20 (100%)	0	<0.001
CAG with IM	10	7 (70%)	3 (30%)	
IM beside GC	20	9 (45%)	11 (55%)	
Dysplasia	10	4 (40%)	6 (60%)	
GC	157	15 (9.6%)	142 (90.4%)	

#: Linear-by-Linear Association chi-square test

CAG: Chronic atrophic gastritis without intestinal metaplasia and dysplasia; IM: Intestinal metaplasia; GC: Gastric carcinoma.

LAPTM4B: Lysosomal-associated protein transmembrane-4 beta

### Expression of LAPTM4B-35 in gastric carcinoma tissues by Western blot analysis

**Fig 2:**
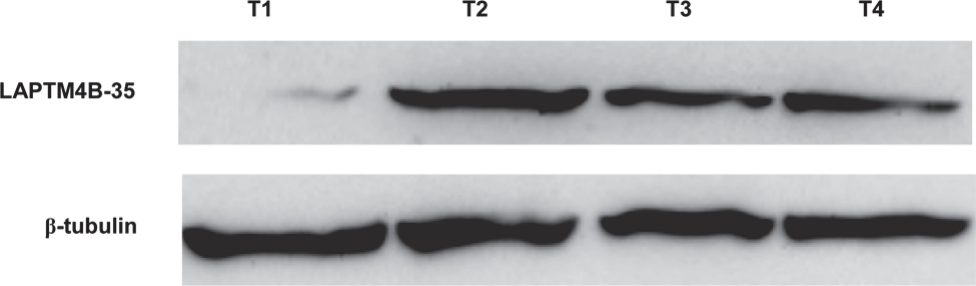
LAPTM4B-35 expression in gastric carcinoma tissues by Western blotting analysis. Protein samples obtained from frozen gastric carcinomas were analyzed by SDS-PAGE followed by immunoblotting with LAPTM4B-N1–99-pAb. In all samples, LAPTM4B-35 protein was detected as an approximately 35-kD band (upper panel). The levels of β tubulin were used as an internal control (lower panel).

Western blot analysis was performed to investigate LAPTM4B-35 protein expression in four frozen GC tissue samples. In all samples, LAPTM4B-35 protein was detected as an approximately 35-kD band (Fig. 2). The expression intensity of LAPTM4B-35 protein observed in Western blot analysis using fresh tissue was consistent with the staining score in immunohistochemical analysis using paraffin section from the same patient.

### Expression of LAPTM4B-35 was in relation to clinicopathologic features of gastric carcinoma

The negative and positive expression categories and the clinicopathologic characteristics of GC in two cohorts were summarized in Table 1 and Table 2. Positive LAPTM4B-35 expression was more frequent detected in cases with distant metastasis (*P* = 0.023) and III+IV TNM stages (*P* = 0.042) in discovery cohort. However, such association was not observed in the testing cohort. No significant relationship was found between the expression of LAPTM4B-35 and the age, sex, tumor size, TNM stage, Lauren type, vessel permeation and lymph nodes metastasis in two cohorts.

### Univariate analysis of prognostic impact of LAPTM4B-35 expression on overall survival in gastric carcinoma

**Fig 3:**
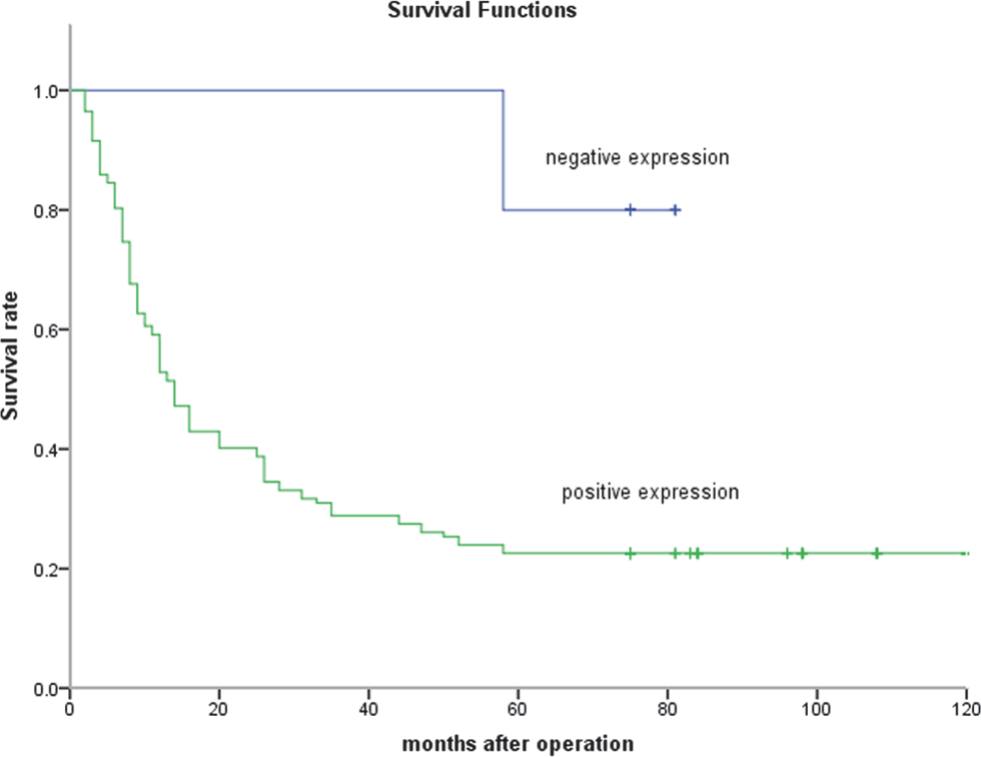
Kaplan-Meier survival curves for discovery cohort with negative or positive LAPTM4B-35 expression. The survival of patients with negative LAPTM4B-35 expression was significantly longer than that of patients with positive LAPTM4B-35 expression; log-rank value = 16.776, *P* < 0.001.

**Fig 4:**
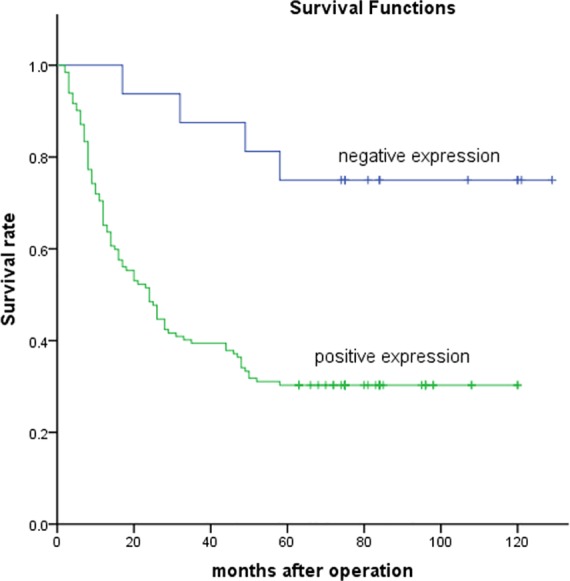
Kaplan-Meier survival curves for testing cohort with negative or positive LAPTM4B-35 expression. The survival of patients with negative LAPTM4B-35 expression was significantly longer than that of patients with positive LAPTM4B-35 expression; log-rank value = 10.624, *P* = 0.001.

Patients with LAPTM4B-35 positive expression had significantly lower 5-year overall survival rate than those with negative expression in both the discovery cohort (32/142 vs. 12/15, *P*<0.001) and the testing cohort (40/132 vs. 12/16, *P*<0.001). Kaplan-Meier survival curves showed that LAPTM4B-35 was the significant variable in the univariate analysis for the impact of LAPTM4B-35 expression on overall survival in discovery cohort and testing cohort (two-sided log-rank test, *P* < 0.001 and *P =* 0.001 respectively; Fig. 3 and Fig. 4).

### Multivariate analysis of prognostic impact of LAPTM4B-35 expression on overall survival in gastric carcinoma

To analyze the independent impact of LAPTM4B-35 expression on overall survival, multivariate analysis by Cox proportional hazards regression model was performed (backward, stepwise). The results revealed that LAPTM4B-35 expression was independent prognostic indicator for the overall survival in patients with GC in both cohorts. The results were presented in Table 4 and Table 5.

**Table 4:** Multivariate analysis of the prognostic factors in patients with gastric carcinoma by Cox proportional hazard model in discovery cohort.

Variables	B	SE	RR (95% CI)	*P* Value#
LAPTM4B-35 expression				
+ versus -	2.205	0.605	9.066 (2.771–29.668)	<0.001
Metastasis				
Present versus Absent	1.217	0.245	3.376 (2.087–5.459)	<0.001
Differentiation				0.005
Moderate versus Well	1.028	0.379	2.796 (1.330–5.881)	0.007
Poor versus Well	0.884	0.358	2.421 (1.199–4.886)	0.014
Sex				
Female versus Male	1.141	0.249	3.131(1.922–5.102)	<0.001
Vessel permeation				
Present versus Absent	0.652	0.292	1.919(1.083–3.399)	0.025
Lymph nodes metastasis				
Present versus Absent	1.620	0.508	5.055(1.869–13.670)	0.001

LAPTM4B: Lysosomal-associated protein transmembrane-4 beta

B: coefficient; SE: standard error; RR: relative risk; CI: confidence interval

#: Cox regression test.

**Table 5:** Multivariate analysis of the prognostic factors in patients with gastric carcinoma by Cox proportional hazard model in testing cohort.

Variables	B	SE	RR (95% CI)	*P* Value#
LAPTM4B-35 expression				
+ versus -	1.505	0.516	4.503 (1.638–12.383)	0.004
Metastasis				
Present versus Absent	0.822	0.277	2.274 (1.321–3.916)	0.003
Sex				
Female versus Male	0.540	0.216	1.715(1.124–2.617)	0.012
TNM stage				
III+IV versus I+II	1.620	0.508	5.055(1.869–13.670)	0.001

LAPTM4B: Lysosomal-associated protein transmembrane-4 beta

B: coefficient; SE: standard error; RR: relative risk; CI: confidence interval

#: Cox regression test.

## Discussion

In the present research, we found that the expression of LAPTM4B-35 was increased steadily in sequential stages of gastric carcinogenesis. No expression of LAPTM4B-35 was found in normal gastric mucosa and chronic atrophic gastritis without intestinal metaplasia and dysplasia. The LAPTM4B-35 expression rate was 30% in chronic gastritis with intestinal metaplasia, 55% in intestinal metaplasia beside cancer tissues, 60% in dysplasia, peaking at 90.4% in GC (Table 3).

The precise mechanism underling gastric carcinogenesis was not yet fully understood. Gastric carcinoma especially ‘Intestinal-type’ carcinoma was developed from sequential stages: chronic gastritis, chronic atrophic gastritis, intestinal metaplasia, dysplasia and gastric carcinoma [28]. Intestinal metaplasia and dysplasia were closely correlated with risks of GC in the multistep process of gastric carcinogenesis [29]. To elucidate molecular and genetic alterations underlying the progression of precancerous gastric lesions might guide us to find markers, such as expression of c-Met [30] and p16 methylation [31], which were valuable in predicting progression of precancerous gastric lesions. Previous reports indicated that LAPTM4B gene over-expression increased cell growth and proliferation [20, 21, 22, 23]. Our present research suggested that LAPTM4B might play important roles in the initial stages of gastric carcinogenesis and was ongoing work continuously. The increased expression of LAPTM4B-35 may predict the progression of gastric lesions and guide doctors to take appropriate interventions slowing the progression of precancerous gastric lesions and reducing the incidence of gastric cancer.

We also found that positive expression of LAPTM4B-35 in GC was more frequent detected in cases with distant metastasis (*P* = 0.023) and III+IV TNM stages (*P* = 0.042) in the discovery cohort. The present work was consistent with previous studies in carcinomas derived from breast [7], pancreas [8], ovary [9, 10, 11], colon [12], liver [13, 14, 15], extrahepatic bile duct [16], cervix [17], endometrium [18] and gallbladder [19]. Carcinomas with LAPTM4B-35 over-expression may present more invasive characteristics. Several studies performed in cell lines demonstrated that over-expression of LAPTM4B-35 promoted the progression of cancer cells toward highly invasive and metastatic stages via mechanisms involving activation of proto-oncogenes such as c-myc, c-fos and c-jun, upregulation of cell cycle regulators such as cyclin D1 and cyclin E [21, 22], resistance to apoptosis, activation of PI3K/AKT signaling pathway [23] and modulating molecules associated with degradation of extracellular matrix [26].

Moreover, our data also demonstrated that the GC patients with positive LAPTM4B-35 expression had significantly lower 5-year overall survival rate than those with negative expression. Multivariate analysis demonstrated that LAPTM4B-35 expression was an independent prognostic factor for overall survival in patients with GC. In previous studies, the same conclusions were got that LAPTM4B-35 was an independent prognostic factor in a wide range of carcinomas [7–19]. LAPTM4B-35 was over-expressed in most human malignant tumors and precancerous lesions. Hence, we believed that LAPTM4B played important roles in the initiation, progression and metastasis of tumors. In fact, many researchs provide various markers in blood or body tissues for diagnosis, prognosis and therapeutics of GC. Such as CD133[32], matrix metalloproteinase-9 [33], c-Met[34], et al. We hope to generate accurate diagnoses, prognoses and select the most appropriate therapy based on the markers. However, there are no current excellent markers for GC. Determining the significance of the markers requires an accumulation of practice.

Recent studies showed that LAPTM4B had critical roles in multidrug resistance (MDR) via increasing drug efflux by P-glycoprotein (P-gp) to reduce drug concentration in cytoplasm and entry into nucleus, decreasing drug-induced DNA damage and escaping from drug-induced apoptosis [35, 36]. It was therefore possible that LAPTM4B-35-knockdown by RNAi may provide a promising novel therapy strategy in LAPTM4B-35 over-expression GC or other cancers.

Recent studies showed that there were two alleles of the LAPTM4B gene, named as LAPTM4B*1 and LAPTM4B*2. The allele *2 of LAPTM4B was found to be the risk factor of the certain tumors, such as breast carcinoma [37], colon carcinoma [38], hepatocellular carcinoma[39] and gastric carcinoma [40], and LAPTM4B *2 was associated with poor prognosis of breast carcinoma [37], colon carcinoma [38] and hepatocellular carcinoma[39]. Although the exact molecular mechanisms underlying the function of LAPTM4B in carcinogenesis had not as yet been worked out, LAPTM4B*2 may be an oncogene or play an important role in cell cycle control. Therefore, further elucidation of the role of LAPTM4B gene in the GC will require genotyping in additional samples in the future studies.

In conclusion, the present research demonstrated that LAPTM4B-35 was over-expressed in a large proportion of patients with precancerous gastric lesions or GC. There were steadily increasing expression of LAPTM4B-35 in series of precancerous gastric lesions. And a close association between LAPTM4B-35 over-expression and disease progression, poor prognosis in GC was found for the first time. These findings suggested that LAPTM4B may be a useful and critical molecular marker to predict the progression of precancerous gastric lesions and the prognosis of patients with GC. LAPTM4B gene may provide a useful target of interventions slowing the progression of precancerous gastric lesions and a new therapy method to improve the prognosis of patients with GC.
